# Profiling of RNA Degradation for Estimation of *Post Morterm* Interval

**DOI:** 10.1371/journal.pone.0056507

**Published:** 2013-02-20

**Authors:** Fernanda Sampaio-Silva, Teresa Magalhães, Félix Carvalho, Ricardo Jorge Dinis-Oliveira, Ricardo Silvestre

**Affiliations:** 1 Institute for Molecular and Cell Biology - IBMC, University of Porto, Porto, Portugal; 2 Department of Sciences, Advanced Institute of Health Sciences North, CESPU, CRL, Gandra, Portugal; 3 Department of Legal Medicine and Forensic Sciences, Faculty of Medicine, University of Porto, Porto, Portugal; 4 Center of Forensic Sciences (CENCIFOR), Portugal; 5 REQUIMTE, Laboratory of Toxicology, Department of Biological Sciences, Faculty of Pharmacy, University of Porto, Porto, Portugal; 6 Department of Diagnostic and Therapeutic Technologies, Polytechnic Health Institute North, CESPU, CRL, Vila Nova de Famalicão, Portugal; 7 National Institute of Legal Medicine and Forensic Sciences, North Branch, Porto, Portugal; 8 Biomedical Sciences Institute “Abel Salazar”, University of Porto, Porto, Portugal; Erasmus University Medical Center, The Netherlands

## Abstract

An estimation of the *post mortem* interval (PMI) is frequently touted as the Holy Grail of forensic pathology. During the first hours after death, PMI estimation is dependent on the rate of physical observable modifications including *algor*, *rigor* and *livor mortis*. However, these assessment methods are still largely unreliable and inaccurate. Alternatively, RNA has been put forward as a valuable tool in forensic pathology, namely to identify body fluids, estimate the age of biological stains and to study the mechanism of death. Nevertheless, the attempts to find correlation between RNA degradation and PMI have been unsuccessful. The aim of this study was to characterize the RNA degradation in different *post mortem* tissues in order to develop a mathematical model that can be used as coadjuvant method for a more accurate PMI determination. For this purpose, we performed an eleven-hour kinetic analysis of total extracted RNA from murine's visceral and muscle tissues. The degradation profile of total RNA and the expression levels of several reference genes were analyzed by quantitative real-time PCR. A quantitative analysis of normalized transcript levels on the former tissues allowed the identification of four quadriceps muscle genes (*Actb*, *Gapdh*, *Ppia* and *Srp72*) that were found to significantly correlate with PMI. These results allowed us to develop a mathematical model with predictive value for estimation of the PMI (confidence interval of ±51 minutes at 95%) that can become an important complementary tool for traditional methods.

## Introduction

The *post mortem* interval (PMI) describes the period of time elapsed from the time of death. Estimation of the PMI is a major focus of investigation in criminal, civil and forensic sciences. In spite of extensive literature on this topic for more than a century, PMI estimation still remains difficult, even for experienced pathologists [1]. All methods presently used to estimate the time of death are affected by some degree of inaccuracy. They only provide a mere approximation since several variables are present (environment temperature, body structure, cause of death, location of the body or putative drug consumption), which can influence the rate of the *post mortem* changes [1]. Currently, there are multiple approaches for estimation of PMI that incorporate methods from almost every discipline of forensic sciences. Among these, forensic pathologists commonly assess physical (*algor mortis*, *livor mortis*), physicochemical (*rigor mortis*), biochemical (electrolyte concentration, enzyme activity), microbiological (decomposition), entomological and botanical processes [2], [3]. Most of these methods for the estimation of PMI remain relatively inaccurate, and even when applied to the very early *post mortem* period are of limited practical relevance [1], [4]. Novel methods such as flow cytometry, capillary zone electrophoresis, magnetic resonance spectroscopy and immunohistochemistry have been proposed to assess *post mortem* changes, attempting to extrapolate the time since death [5]. Although these approaches do not make on their own death time estimation more precise, the combination of different methods has been proposed to narrow down the margins of error associated to individual methods [1], [4], [5].

An accurate estimation of the PMI requires the evaluation of parameters that change constantly with time after death. This definition seems to fit well in *post mortem* degradation of nucleic acids [6]. Indeed, with the advances of molecular biology, the analysis of time-dependent degradation of nucleic acids (both DNA and RNA) became a focus of attention in clinical medicine as well as in forensic science [6], [7]. In this context, the RNA potential has been studied for several purposes, namely the identification of body fluids [8], [9], [10], time-dependent expression of troponin I mRNA in the contused skeletal muscle as a possible marker for wound age estimation [11], [12], and *post mortem* assessment of the functional status of cells and organs aiming the diagnosis of cause and mechanism of death [13]. The estimation of the PMI by studying the RNA decay may be within reach since RNA degradation or loss of RNA transcripts after death seems to be rapid and time-dependent [14]. After death RNA is degraded by ribonucleases already present in the cell and/or originating from bacteria or other environmental contamination. Physical and unspecific chemical factors add to this effect. The concept of quantification of mRNA or DNA degradation as a possible indicator of *post mortem* interval has already been presented by some authors [7], [11]. Intriguingly and despite its huge potential, few studies were able to correlate the degradation of defined RNA transcripts with PMI. Therefore, this study aims to define a panel of RNA transcripts, in different biological matrices, by which *post mortem* degradation may be used to significantly correlate with PMI. Subsequently, the combined quantification of the previously defined transcripts was used to develop a mathematical model for PMI estimation, which provides a serious advantage to currently available methodology that can be used as a coadjuvant approach to increase the accuracy of PMI estimation.

## Materials and Methods

### Samples

#### Preliminary experiments

Balb/c mice (n = 5) were euthanized by isoflurane anesthesia followed by cervical dislocation. Eight organs (skin, heart, spleen, femoral quadriceps, liver, pancreas, stomach and lungs) were collected following defined guidelines to obtain *post mortem* specimens for forensic applications [15]. Identical pieces (10–70 mg) of each organ were immediately separated from mouse corpse after euthanasia, transferred into eppendorf tubes and maintained in temperature (21°C) and sterility controlled conditions for 4 or 20 hours *post mortem*.

#### Kinetic analysis

Balb/c mice (n = 15) were euthanized by isoflurane anesthesia followed by cervical dislocation. Three organs (heart, femoral quadriceps and liver) were collected as described above. Briefly, each organ was separated from mouse corpse immediately after euthanasia and was cut into 12 identical pieces (5–20 mg), each corresponding to a time point. The tissues samples were immediately stored (for time 0) or maintained in temperature (21°C) and sterility controlled conditions up to 11 hours.

#### Proof of applicability

Femoral quadriceps tissues were recovered from intact animal corpses (Balb/c mice) at 1, 4 and 10 h after euthanasia (n = 5 for each time point).

At each defined time point, 200 µL of RNA later stabilization reagent (Qiagen®) was added, following with immediately storage at −80°C until RNA extraction. All samples were posteriorly used for both RNA integrity and quantitative PCR analyses. All research was approved by the National Council of Ethics for the Life Sciences (CNECV). Animal experiments were approved by the Portuguese Agency for Animal Welfare (general board of Veterinary Medicine in compliance with the Institutional Guidelines and the European Convention). The corresponding author (Ricardo Silvestre) has an accreditation for animal research given from Portuguese Veterinary Direction (Ministerial Directive 1005/92).

### Total RNA extraction

Total RNA isolation was performed by adding 500 µL of guanidinium thiocyanate-phenol-chloroform extraction method (TRI Reagent, Sigma-Aldrich, St. Louis, MO) to samples. All specimens were homogenized by mechanical disruption using the Ultra-Turrax Mixer (IKA®) instrument and total RNA was extracted in RNAse-free environment and kept at −80°C until use. A DNase digestion step (RNase-Free DNase Set, Qiagen) was included and all RNA was ressuspended with ultrapure water (Qiagen). This ultrapure water was similarly used for all subsequent dilutions of RNA samples.

### Determination of RNA Integrity

Nucleic acid concentrations were measured in a NanoDrop® ND-1000 Spectrophotometer (NanoDrop Technologies, USA). Purity of the total RNA extracted was quantified by measuring the absorbance at 230, 260 and 280 nm. Through the concentration of each sample assessed in NanoDrop®, all samples were diluted to use the same amount of total RNA (200 ng) to assess their quality. RNA quality of each sample was assessed by a microfluidic-based electrophoresis system using the Experion™ Automated Electrophoresis System (Bio-Rad Laboratories, USA), which automatically assesses the integrity of RNA samples expressed as a RNA quality indicator (RQI) number.

### cDNA synthesis

All RNA extracted from murine samples was retrotranscribed to cDNA using the iScript cDNA Synthesis Kit® (Bio-Rad) according to the manufacturer's protocol. For the cDNA synthesis, approximately 1 µg of each sample total RNA were used. Each 20 µL reaction mix contained: 4 µL of 5× iScript reverse transcription supermix, 1 µg of total RNA and RNase-free water to make up 20 µL. The cDNA synthesis was performed in a Mastercycler ep gradient S (Eppendorf®) with following steps: priming at 25°C for 5 min, reverse transcription at 42°C for 30 min and enzyme inactivation at 85°C for 5 min. All cDNA samples were stored at −20°C until quantitative real-time PCR (qPCR) analysis.

### Quantitative real-time PCR

qPCR was performed in IQ™ 5 Real-Time PCR detection System (Bio-Rad) in 96-well plates (Bio-Rad) with a reaction volume of 20 µL and runs up to 40 cycles using iQ™ SYBER® Green Supermix. The final PCR reaction mixture of 20 µL contained 0.5 µL of cDNA sample, 10 µL of iQ™ SYBR® Green Supermix, 0.5 µL of each primer and 8.5 µL of RNase-free water. The cycling conditions were set as follows: Taq DNA polymerase activation at 95°C for 3 min, amplification steps: denaturation at 95°C for 15 s, annealing at 60°C for 15 s, and extension at 72°C for 15 s with fluorescence acquisition. The cycling conditions were equal for all genes except for the *Bhmt* gene, which has an annealing temperature of 64°C. Genomic DNA contamination was verified by reaction without reverse transcription in randomly tested samples, which resulted in Ct values >38. Based on literature, we have chosen for our analysis 11 genes (Table 1). Five commonly used reference genes were chosen (*Actb*, *Gapdh*, *Hprt*, *Cyp2E1* and *Ppia*) as widely used stable expressed genes [16], [17]. *Alb*, *Bhmt*, *Mylk* and *Tpm1* were chosen as chosen as being the most expressed tissue-specific genes [18]. We also included in our panel the 40S ribosomal protein S29 (*Rps29*) and the 72 kDa subunit of the signal recognition particle (*Srp72*) genes, which proved to be more stable than other commonly used reference genes [19], [20]. Details on the measurements of all this transcripts are provided in Figures S1A and S1B. All cDNA samples were measured in duplicate, and mean values of the quantification cycle Ct were used for calculations. Data were normalized according to the ΔCt model with the following formula: ΔCt = Ct _(target gene)_−Ct _(reference gene)_. Gene expression changes were analyzed using the built-in iQ5 Optical system software (version 2). All real time experiments were performed following the recently defined Quantitative Real-Time PCR Experiments (MIQE) guidelines [21]. Details are provided in Table S1. All primers (HPLC purified, Stabvida) were designed using Beacon Designer software (version 7.2, PREMIER Biosoft International, Palo Alto, CA) and thoroughly tested.

**Table 1:** Murine genes targeted for quantitative analysis.

Gene Symbol	Accession number	Gene Name	Function
***Tpm1***	NM_024427.4	Tropomyosin-1	Cell structure
***Alb***	NM_009654.3	Serum albumin	Transport protein
***Actb***	NM_007393.3	Beta actin	Cytoskeletal structural protein
***Gapdh***	NM_008084.2	Glyceraldehyde-3-phosphate dehydrogenase	Involved in glycolysis
***Hprt***	NM_013556.2	Hypoxanthine-guanine phosphoribosyltransferase	Purine synthesis in salvage pathway
***Ppia***	NM_008907.1	Cyclophilin A	Involved in signal transduction
***Bhmt***	NM_016668.3	Betaine–homocysteine S-methyltransferase 1	Amino acid metabolism
***Srp72***	NM_025691.1	Signal recognition particle 72	Protein synthesis
***Cyp2E1***	NM_021282.2	Cytochrome P450 2E1	Oxidative metabolism
***Mylk***	NM_139300.3	Myosin light-chain kinase	Mechanism of contraction in muscle
***Rps29***	NM_009093.2	40S ribosomal protein S29	Protein synthesis

### Data analysis

Quantification and expression data were statistically processed using GraphPad Prism 5. The determined *p* values of the statistical significance were examined using linear regression and the Pearson correlation (r). An acceptable Pearson correlation value of r>0.900 was considered for posterior analysis. Mathematical model was developed from the 11 hour kinetics, by performing regression analysis using Tool Analysis supplement of Microsoft Excel 2010 for Windows, in order to estimate the slope (m) and *y*-intercept (b) errors. Level of significance was always set to *p*<0.05.

## Results

### Loss of RNA integrity during PMI is tissue-specific

**Figure 1:**
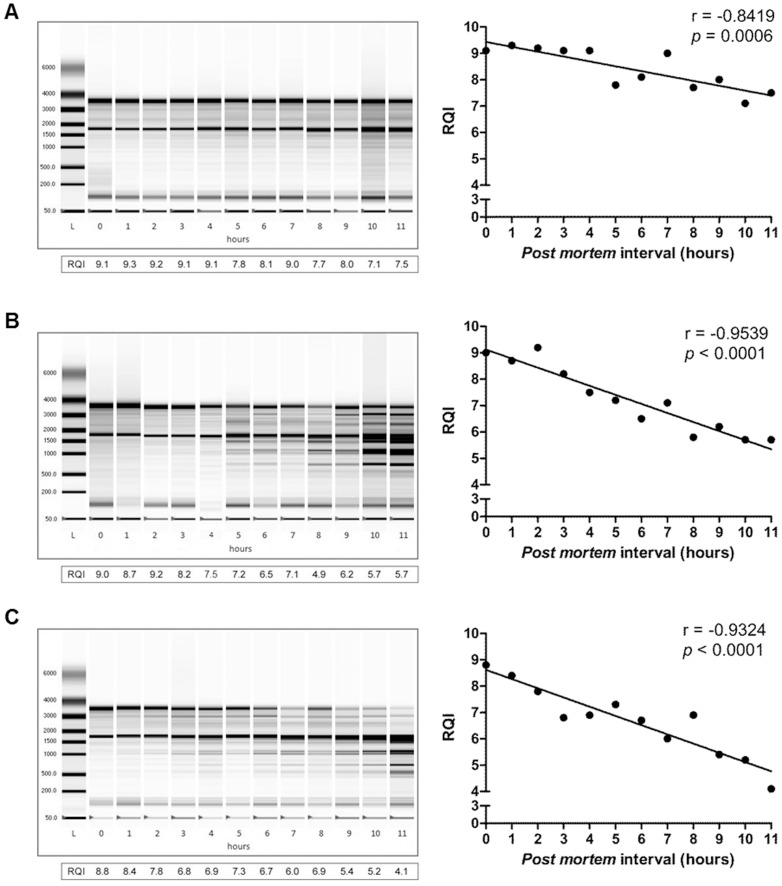
Loss of RNA integrity from heart, femoral quadriceps and liver tissue samples significantly correlate with the *post mortem* interval. The degradation profile of total RNA recovered from heart (A), femoral quadriceps (B) and liver (C) tissue samples was evaluated at different time periods *post mortem* (0–11 h) by RNA electrophoresis (Experion®). For each tissue, the RNA quality indicator (RQI) is plotted against the *post mortem* interval (L – single strand RNA ladder). Pearson correlation (r) and *p* value are depicted for each organ. One representative experiment out of 15 is shown.

The enormous potential of RNA technologies, together with reports of unexpectedly high stability in certain conditions, have stimulated forensic researchers to explore the RNA world [14]. Among its potentialities, time dependent RNA decay analysis was suggested to assess the PMI [7], [22]. Nevertheless, the integrity of RNA among different *post mortem* tissues or organs is known to behave differently [23], [24] adding a new level of complexity to such analysis. Our strategy began by analyzing the tissue-specificity of RNA integrity loss during PMI. One has to be conscious of the existence of several parameters that may influence the rate of RNA decay in *post mortem* organs. Environmental temperature and microbiological contamination are among the most relevant variables. Since we aimed to define a panel of RNA transcripts that significantly correlate with PMI, it was mandatory to experimentally control these two parameters. Therefore, immediately after euthanasia, samples of the 8 murine tissues; heart, lung, spleen, femoral quadriceps, liver, stomach, pancreas and skin were removed and maintained in aseptic conditions at controlled temperature (21°C). Four and twenty hours *post mortem*, the extracted RNA was evaluated for purity and integrity. These organs were chosen due to its high potential in forensic area and their applicability for gene expression studies using *post mortem* human tissue [15], [23], [25], [26], [27]. As a method for RNA quality assessment, we determined the RNA Quality Indicator (RQI) that quantifies the integrity of the RNA samples. According to the RQI values obtained, three different groups were formed (Figure S2). The first group (I) was constituted by heart, spleen and lung, which displayed the highest stability, even for longer PMI, maintaining RQI values above 6. The group II was composed by the femoral quadriceps, liver and stomach. The RNA recovered from these organs has a faster degradation rate, as observed with the time dependent decrease of the RQI values. Pancreas and skin composed the third group (III), where low RNA integrity (RQI below 4) was observed since represent ribonuclease-rich organs [28]. This confirms the tissue specificity for correlative analysis between time and RNA degradation. Based on these results, we pursued our study by performing kinetic analysis during an eleven hour period (checkpoint at every single hour) of representative organs of group I and II (heart, femoral quadriceps and liver). Accordingly some authors, to minimize the impact of RNA integrity, only RQI values above 5 are acceptable for successful and reliable qPCR quantification [26], [29], [30], which led us to exclude the third group from the study. RNA recovered from the heart demonstrated to be extremely stable *post mortem*, as shown by RQI values above 7 even after 11 h (Fig. 1A). This reflects the quality of this tissue for PMI assessment. Remarkably, no significant differences were observed in the RQI levels during the first 4 hours *post mortem*, although a time dependent decrease in RNA integrity was observed until 11 hours *post mortem*. Indeed, RNA from heart tissue samples showed a significant correlation (*p* = 0.0006) with the PMI until 11 h. In opposition to heart tissue, *post mortem* RNA recovered from both femoral quadriceps (Fig. 1B) and liver (Fig. 1C) immediately began to decay. Nevertheless, the loss of RNA integrity in both tissues significantly correlated with PMI (*p*<0.0001) with a Pearson correlation higher than 0.900.

### Femoral quadriceps mRNA transcripts degradation significantly correlates with PMI

**Figure 2:**
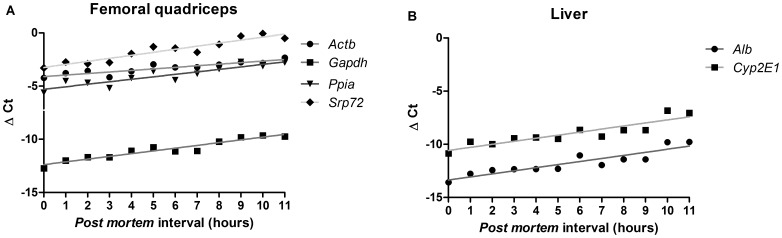
Six genes (*Alb*, *Actb*, *Gapdh*, *Ppia*, *Cyp2E1* and *Srp72*) in two tissues (femoral quadriceps and liver) identified to significantly correlate with the *post mortem* interval. The normalized expression levels of these genes analyzed by quantitative real time PCR is plotted against the PMI in the femoral quadriceps (A) and liver (B). One representative experiment out of 15 is depicted.

To evaluate RNA decay over the PMI, we have defined a list of eleven genes for quantitative PCR analysis (Table 1). Based on the knowledge that gene transcription is tissue-selective and context-dependent a panel of both evolutionarily-conserved (*Rps29* and *Srp72*) [19], [20], most expressed tissue-specific mRNA (*Bhmt* and *Alb* for the liver and *Tpm1* and *Mylk* for the femoral quadriceps and cardiac muscle) and more ubiquitous transcripts (*Actb*, *Gapdh*, *Hprt*, *Ppia* and *Cyp2E1*) were chosen. The threshold cycle (Ct) value is defined as the point at which fluorescence rises above the background fluorescence. The Ct values and thus the copy number of template molecules varied among the transcripts and PMI (Table S2). To eliminate this potential bias and to control the variations in extraction or reverse transcription yield and efficiency of amplification, we have normalized all target genes against *Rps29*, which was chosen taking into account its high degree of stability. Indeed, we observed that the transcription of this gene showed the lowest variations in the gene expression between all PMIs and organs. As an example, the coefficient of variation (CV) for the Ct values obtained for *Rps29* transcripts on all femoral quadriceps samples was lower than 1% (ranging from 25.4 to 26.0), while it varies between 2.7–5.5% for all the other analyzed transcripts. After normalization, the Pearson correlation (r) and *p* value were assessed for each gene within each organ (Table 2). Interestingly, no significant correlation was found between any analyzed transcript in heart samples and PMI. The ΔCt values were found constant for all genes in all PMIs (Table S3), although the heart RNA integrity was found to linearly decrease with time (Fig. 1A). On the other hand, the decay of several femoral quadriceps and liver transcripts were found to positively correlate with the PMI (Table 2) and ΔCt values showed greater variation (Tables S4, S5, S6, S7). Aiming to assure the maximum significance between gene decay and PMI only those genes with Pearson correlations higher than 0.900 and found to be statistically significant were considered for posterior analysis (Fig. 2 and represented in bold in Table 2). This approach allowed the identification of 4 genes (*Actb*, *Gapdh*, *Ppia* and *Srp72*) in the femoral quadriceps and two genes (*Alb* and *Cyp2E1*) in the liver as the most reliable dependent variables that correlate with PMI. The need for method uniformization and systematization, accompanied with narrow confidence intervals led us to focus the construction of a mathematical model on the transcripts identified in the femoral quadriceps, which displayed the larger number of correlational variables.

**Table 2:** Pearson correlation (r) and *p* values of linear regression for each gene in the tested organs.

Gene	Heart		Femoral quadriceps		Liver	
	r	*p*	r	*p*	r	*p*
***Tpm1***	−0.393	0.205	0.194	0.5440	0.711	0.0095
***Alb***	0.456	0.135	0.862	0.0003	**0.913**	**<0.0001**
***Actb***	0.439	0.152	**0.910**	**<0.0001**	0.865	0.0003
***Gapdh***	0.359	0.251	**0.958**	**<0.0001**	0.895	<0.0001
***Hprt***	0.219	0.493	0.881	0.0002	0.256	0.4203
***Ppia***	−0.199	0.533	**0.909**	**<0.0001**	0.855	0.0004
***Bhmt***	–	–	–	–	0.842	0.0006
***Srp72***	0.189	0.554	**0.947**	**<0.0001**	0.824	0.0010
***Cyp2E1***	0.341	0.276	0.608	0.0350	**0.905**	**<0.0001**
***Mylk***	0.498	0.099	0.819	0.0010	0.878	0.0002

Bold highlights Pearson correlations between gene decay and PMI higher than 0.900 and found to be statistically significant.

### Development of a mathematical model with predictive value for PMI estimation

**Figure 3:**
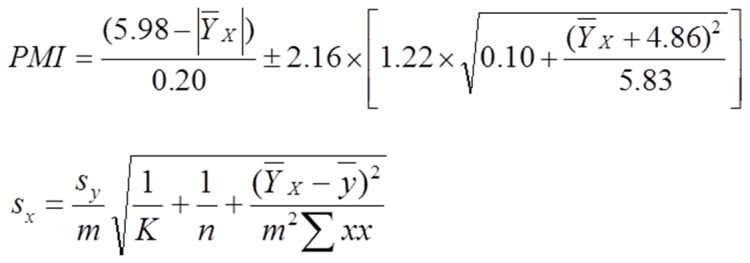
A mathematical model for the accurate estimation of the PMI. The two equations represent the formula to calculate the PMI with a confidence interval of 95% (A) and the standard deviation (B), where (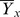) is the average signal for our sample, *K* is the number of replicate samples used to establish 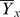, *n* is the number of measured endpoint times, 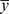 is the average signal for each endpoint time, 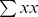 is the summation of (individual *x*−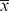)^2^.

The ultimate goal of this study was to develop an abstract model using mathematical language to describe the behavior of gene transcripts decay over PMI. In that sense, we have performed regression analysis with the objective of building descriptive mathematical laws to be used by forensic experts for valid and reliable determination of the PMI. The linearity of the method was determined by evaluation of the regression curve (ΔCt *versus* time) and expressed by the correlation coefficient (*r*). We used three independent calibration curves (y = mx+b) for each of the 4 selected genes to obtain the mean slopes (m) and y-intercept (b). Linearity was accepted if *r*≥0.900. The goal of linear regression was to determine the best estimates for the slope and y-intercept. This was accomplished by minimizing the residual error between the experimental *y* values, and those values predicted by regression line equation. The most commonly used form of linear regression is based on three assumptions: (1) that any difference between the experimental data and the calculated regression line is due to indeterminate errors affecting the *y* values, (2) that these indeterminate errors are normally distributed, and (3) that the indeterminate errors in *y* do not depend on the value of *x*. The derivation of equations for calculating the estimated slope and *y*-intercept can be found elsewhere [31]. This allows us to define a regression equation to calculate the PMI (Fig. 3A). Once known, it is possible to determine the PMI and to estimate the error associated to time. For that, we measured an average signal for our sample (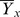) and use it to calculate the value of *x*. The standard deviation for the calculated value of *x (S_x_)* is given by the equation in Fig. 3B, where K is the number of replicate samples (K = 15) used to establish 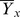, *n* is the number of measured endpoint times (n = 12), 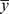 is the average signal for each endpoint time, 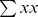 is the summation of individual (*x*−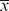)^2^. Once *S_x_* is known, the confidence interval for the PMI can be calculated attending the value of *t* determined by the desired level of confidence (*p*<0.05) for *n*−2 degrees of freedom. Figure S3 evidence the interval of confidence for each sampled endpoint and it is observed that similar results were obtained during all sample period, giving more relevance to our results. The highest error was registered for time zero, which is explained by inherent difficulty to collect samples instantaneously.

As explained above, our model was tested using controlled environmental conditions. We decided to challenge our system by sampling femoral quadriceps tissues from intact mouse corpses at 1, 4 and 10 h after death. RQI values ≥7.9 were obtained. The PMI was calculated using the equation shown in Fig. 3A, substituting the 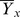 by the average signal of the replicates for each time point (K = 5) and considering ΔCt values for all 4 transcripts (*Actb*, *Gapdh*, *Ppia* and *Srp72*). Remarkably, the medium PMI values obtained were 1.90±0.01, 4.10±0.87 and 9.80±1.87 hours, demonstrating the high predictive power of our mathematical model.

## Discussion

*Post mortem* degradation of nucleic acids has been suggested as an elegant alternative to classical methods for PMI estimation [7]. We hypothesized that a wider analysis of multiple organ and transcript targets would allow us to define the parameters that correlate with PMI in order to develop a mathematical model with predictive value for the accurate determination of the PMI. One of the major problems concerning human tissue samples is the fact that one has to deal with rather heterogeneous sample populations since *ante* and *post mortem* parameters cannot be controlled and are normally not entirely known [32], [33]. Indeed, besides the endogenous and exogenous ribonucleases, which are omnipresent and are found to be responsible for the fast *in vivo* RNA degradation, environmental and storage chemical and thermal conditions can also take place immediately after death and thus influence RNA [34]. Bauer *et al.* [7] attempted to establish the time of death through the quantitative analysis of mRNA degradation by multiplex-qPCR in combination with laser-induced fluorescence capillary electrophoresis. Their results showed a significant correlation between RNA degradation and PMI in stored refrigerated human blood and brain samples for up to 5 days [7]. In opposition, it has been reported an absence of significant correlation between mRNA degradation and PMI in human brain tissue [23], [35]. Although controversial, the studies performed so far evaluating quantitatively RNA degradation as a PMI indicator have only focused on a few mRNA transcripts and therefore large confidence intervals have been obtained [7], [11]. In addition, these studies did not contemplate *ante mortem* interfering factors or different tissues. Koppelkamm *et al.* [36] studied the suitability of ten functionally different gene transcripts as endogenous control genes in gene expression studies of *post mortem* human tissue samples. Results appointed the specific cause of death and the type of tissue as major variables on transcript amounts of certain genes commonly used as endogenous control genes.

Our approach allowed us to circumscribe the potential targets to three distinct tissues: heart, liver and femoral quadriceps; narrowing our sampling for transcriptional analysis. Among all the tissues analyzed, only femoral quadriceps and liver tissue were found to be correlated with PMI in the studied period. Nevertheless, our results also suggest that transcript analysis on heart tissue samples could be interesting for longer PMI. Indeed, preliminary experiments conducted in our murine model aiming to study PMI until 98 hours confirmed the potential of cardiac and skeletal muscle for PMI evaluation (data not shown). Nevertheless, previous studies on gene expression in *post mortem* human cardiac, brain and skeletal muscle (iliopsoas) to estimate PMI did not find any correlation between PMI and the gene transcription decay [23], [37].

Eleven genes that code for proteins with distinct biological functions in tissues were selected for our study in order to significantly reduce the chance that genes may be co-regulated, and thus that could be affected at different rates among the distinct types of death (Table 1). Five commonly used reference genes were chosen (*Actb*, *Gapdh*, *Hprt*, *Cyp2E1* and *Ppia*) as widely accepted stable expressed genes [16], [17], [38]. In spite of the original thought of an ubiquitous expression of such genes, several studies already found an existing variation in the expression of commonly used reference genes [25], [36], [39]. The literature data shows that housekeeping gene expression can vary considerably, although occasionally constant in a given cell type or experimental condition [40], [41], [42]. Our approach confirmed these results, since the majority of the target transcripts (*Actb*, *Gapdh*, *Cyp2E1* and *Ppia*) were found to be significantly correlated with PMI in the femoral quadriceps and liver. *Alb*, *Bhmt*, *Mylk* and *Tpm1* were chosen as being the most expressed tissue-specific genes [18]. The first two were selected since they are specific for liver. Indeed, among all the tested tissues, *Bhmt* was only successfully transcribed in liver. In addition, *Alb* was found to be significantly correlated with PMI in this organ. *Mylk* and *Tpm1* were chosen as being highly expressed in muscle tissues (cardiac and quadriceps), but no correlation was found between the degradation of any of these genes and PMI. Finally, the evolutionarily-conserved *Srp72* gene, which was previously identified as a highly stable reference genes for the normalization of qPCR data [20], was surprisingly found to correlate with PMI in the femoral quadriceps. Although several studies [39], [43], [44], [45] claimed difficulties to find an “universal” reference gene with stable expression for PMI analysis in all cell types and tissues, the *Rps29* gene demonstrated an important potential since proved to be stable in all tissues studied (heart, liver and femoral quadriceps) during the evaluated PMI. Indeed, the transcription of *Rps29* showed the lowest variations in the gene expression between all PMIs and organs, enlarging the concept of *Rps29* stability proposed by De Jonge *et al.* [19] to *post mortem* samples.

The results of this study impelled us to develop a mathematical model to describe the behavior of the identified gene transcripts decay over PMI as a valuable tool for forensic pathologists. In order to validate our mathematical model, we applied it using femoral quadriceps specimens recovered from intact mice corpses kept 1, 4 and 10 hours at room temperature, without any control on environmental or microbiological conditions. This approach allows us to verify any potential bias on RNA degradation induced by natural putrefaction. It is important to mention that the proposed model can match a “death by natural causes” and under environmental temperature similar to a laboratory. Nevertheless, it was not impossible to attain all potential variables that may occur after death and therefore this represents a preliminary study that must be further validated with human samples. As referred above, some authors deny a close link between RNA degradation and the PMI [23], [35]. However, other studies performed aiming to simulate forensic conditions demonstrated a clear correlation [7], although data were obtained under well controlled variables (e.g., constant ambient temperature). In addition, the non-systematic nature of RNA degradation might render difficult to obtain consistent conclusions in bodies from real scenes. To increase the potential applicability of our approach, only RQI≥5 were considered acceptable, although lower RQI cut-offs (≥3.95) have been suggested [46].

### Conclusions

Overall, we observed a time and organ dependent decrease of the RQI values. A significant correlation was found between total RNA isolated from the heart, femoral quadriceps or liver samples and the PMI. A quantitative analysis of several transcripts on these organs led to the identification of 4 genes that when analyzed on a quadriceps specimens correlate with PMI. Furthermore, we developed a mathematical model to estimate the PMI with an error mean of 51.4 minutes. We have successfully challenged our method using femoral quadriceps samples recovered from intact mouse corpses. This study may represent a new paradigm to estimate PMI and become a complementary tool for traditional methods, with the ultimate goal to increase the accuracy of the PMI estimation. The suitability of these strategies for *post mortem* human autopsy material as well as the influence of the various parameters (e.g. cause of death, age at death, gender and body mass index, duration of agony, storage conditions of the body) will be addressed in future studies.

## Supporting Information

Figure S1 **Specificity validation of the PCR products.** Agarose gel electrophoresis of qPCR amplicons (A). Melting peak chart from a melting temperature analysis of ten transcripts, *Tpm1* (a), *Alb* (b), *Actb* (c), *Gapdh* (d), *Hprt* (e), *Ppia* (f), *Srp72* (g), *Rps29* (h), *Cyp2E1* (i) and *Mylk* (j) qPCR amplicons with SybrGreen I detection on the IQ™ 5 Real-Time PCR detection System (Bio-Rad) (B).
(DOCX)

Figure S2 **RNA integrity loss during PMI is tissue-specific.** Graphics show the RQI values of tissue samples collected at 4 and 20 hours *post mortem* maintained at 21°C. Three groups were generated on the basis of RNA integrity. Group I comprised the heart, lung and spleen (A). Group II included the liver, femoral quadriceps and stomach (B), while group III was composed by the skin and pancreas (C). One representative experiment out of three is shown.
(DOCX)

Figure S3 **Variation of the error value (Sx) over time.** The confidence interval as determined in fig. 3A was plotted against the *post mortem* interval.
(DOCX)

Table S1 **MIQE Checklist.**
(XLS)

Table S2 **Repeatability (intra-assay variation) of qPCR measurements.**
(DOC)

Table S3 **ΔCq values of each gene for heart samples of 11 h kinetic normalized against reference gene RPS29.**
(DOCX)

Table S4 **ΔCq values of each gene for femoral quadriceps samples of 11 h kinetic normalized against reference gene RPS29.**
(DOCX)

Table S5 **ΔCq values of each gene for liver samples of 11 h kinetic normalized against reference gene RPS29.**
(DOCX)

Table S6 **qPCR target information: Description of the genes, primer and amplicons.**
(DOC)

Table S7 **qPCR Validation.** Standard curves were generated either from cDNAs (for all RNAs) setting the undiluted sample as 1 arbitrary unit. The Cq values were calculated automatically by the iQ5 Optical system software, version 2 using the “second derivative maximum” method. Standard curves: the PCR-efficiency (E = 10^−1/slope^) and the slope, intercept, and error of the regression line as well as the so-called dynamic range and the Cq variation at the lower limit (the endpoint of the dynamic range) were calculated by the iQ5 Optical system software. The presented data was retrieved from heart samples except for BHMT, which was from liver tissue sample.
(DOC)
