# Correction: Profiling of RNA Degradation for Estimation of *Post Mortem* Interval

**DOI:** 10.1371/annotation/cb8b37ac-cbe2-45e3-b9fe-d62e7ced4b25

**Published:** 2013-06-18

**Authors:** Fernanda Sampaio-Silva, Teresa Magalhães, Félix Carvalho, Ricardo Jorge Dinis-Oliveira, Ricardo Silvestre

The word "Mortem" in the title is spelled incorrectly. The correct title is: Profiling of RNA Degradation for Estimation of Post Mortem Interval. The correct citation is: Sampaio-Silva F, Magalhães T, Carvalho F, Dinis-Oliveira RJ, Silvestre R (2013) Profiling of RNA Degradation for Estimation of Post Mortem Interval. PLoS ONE 8(2): e56507. doi:10.1371/journal.pone.0056507 

