# Supplementary material for: Profiling of RNA Degradation for Estimation of Post Morterm Interval
Source: PLoS One. 2013 Feb 20;8(2):e56507. doi: 10.1371/journal.pone.0056507 (PMC3577908; doi:10.1371/journal.pone.0056507)

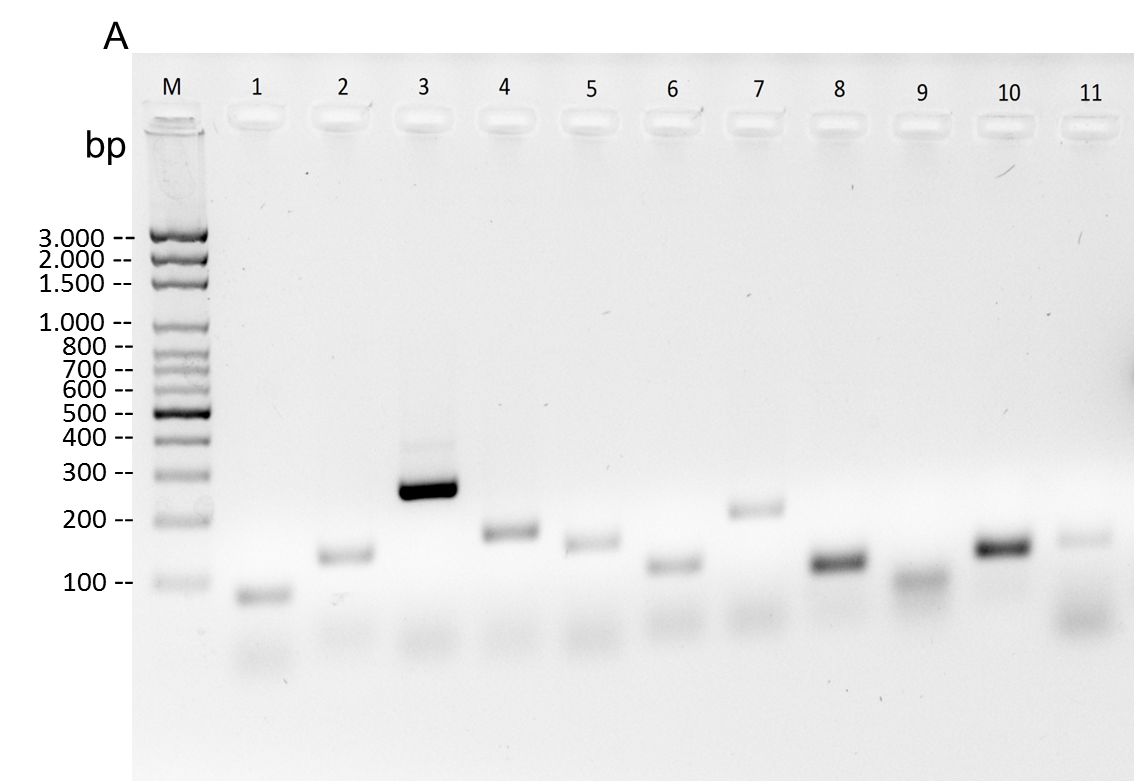

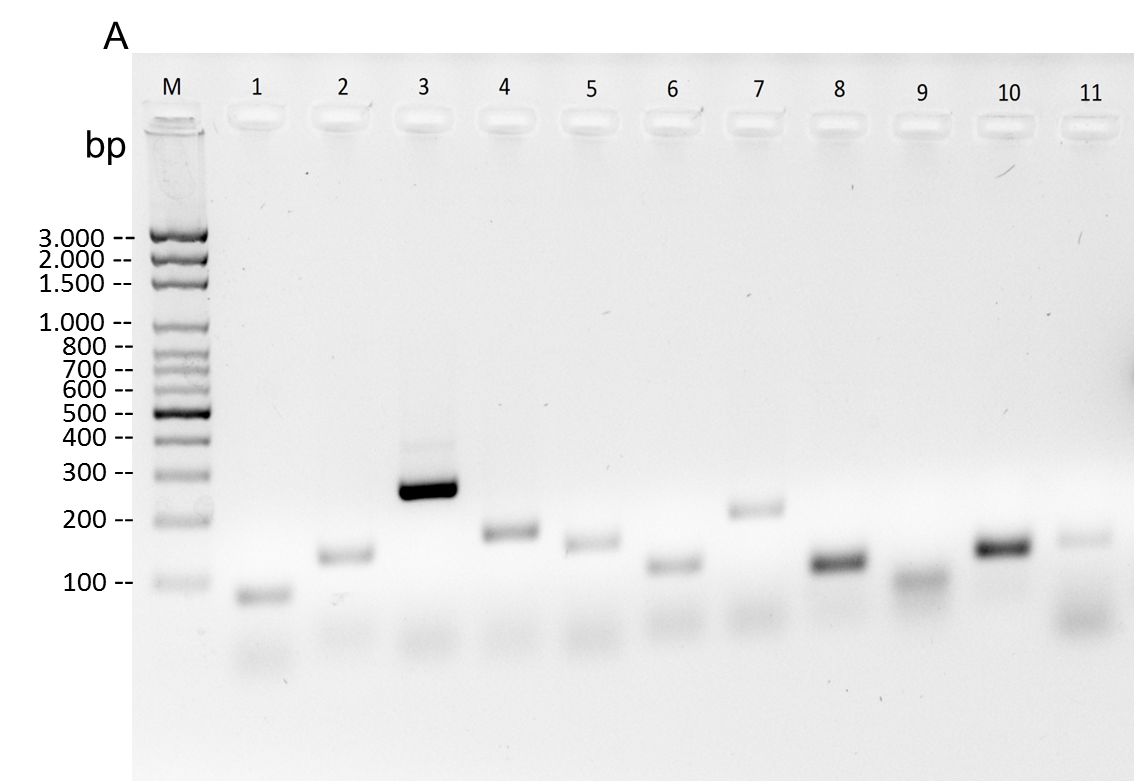


M 1 2 3 4 5 6 7 8 9 10

Lanes and target sizes:

M = 100bp DNA Ladder

(Solis BioDyne, Estonia)

1 = *Tpm1* (82bp)

2 = *Gapdh* (121bp)

3 = *Actb* (274p)

4 = *Alb* (186bp)

5 = *Hprt* (86bp)

6 = *Ppia* (212bp)

7 = *Srp72* (81bp)

8 = *Rps29* (78bp)

9 = *Cyp2E1* (165bp)

10 = *Mylk* (181bp)

bp = base pairs


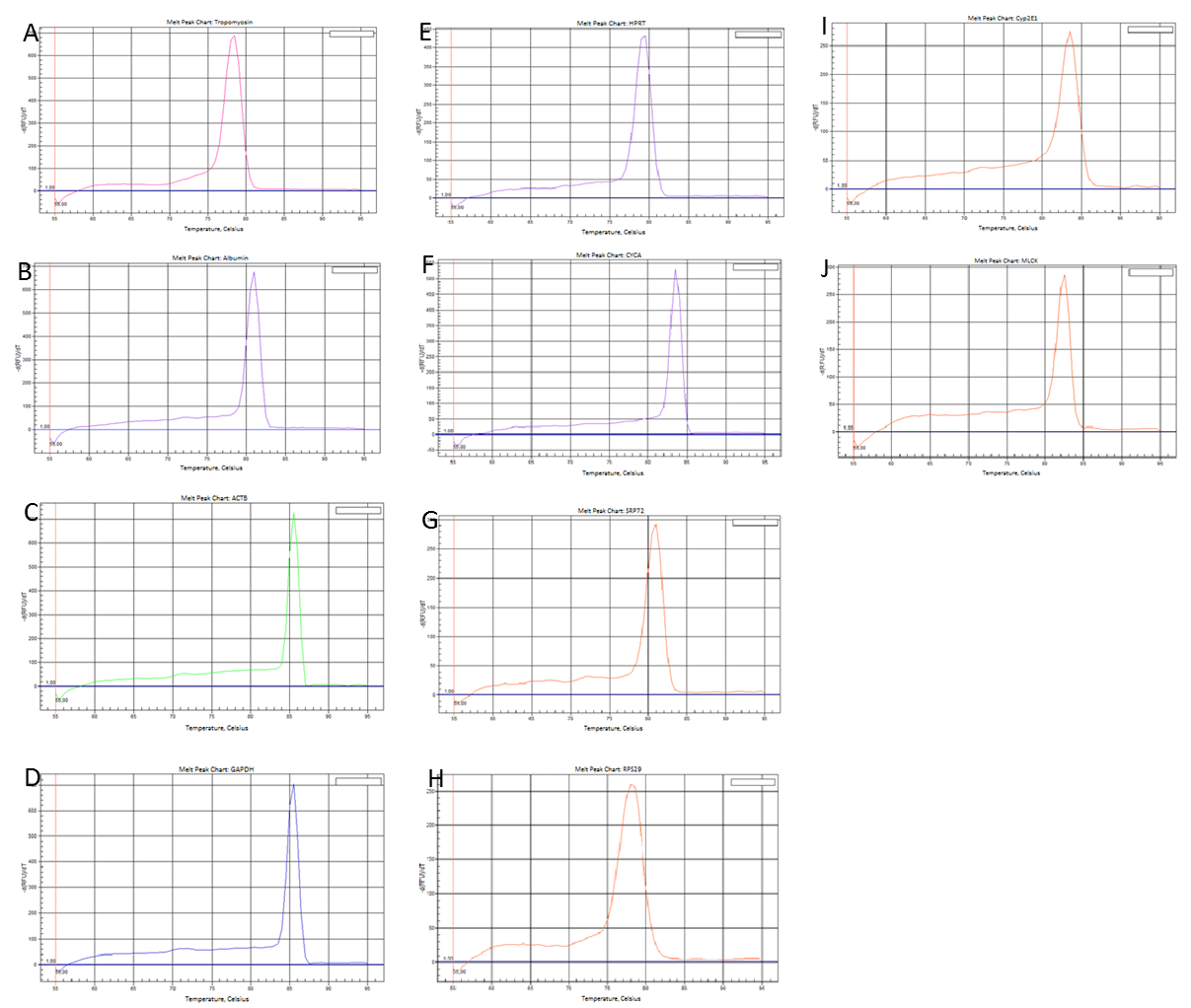

Supplement: Figure S1 — Specificity validation of the PCR products. Agarose gel electrophoresis of qPCR amplicons (A). Melting peak chart from a melting temperature analysis of ten transcripts, Tpm1 (a), Alb (b), Actb (c), Gapdh (d), Hprt (e), Ppia (f), Srp72 (g), Rps29 (h), Cyp2E1 (i) and Mylk (j) qPCR amplicons with SybrGreen I detection on the IQ™ 5 Real-Time PCR detection System (Bio-Rad) (B). (DOCX) [file pone.0056507.s001.docx]
