# Supplementary figures and images for: Profiling of RNA Degradation for Estimation of Post Morterm Interval
Source: PLoS One. 2013 Feb 20;8(2):e56507. doi: 10.1371/journal.pone.0056507 (PMC3577908; doi:10.1371/journal.pone.0056507)

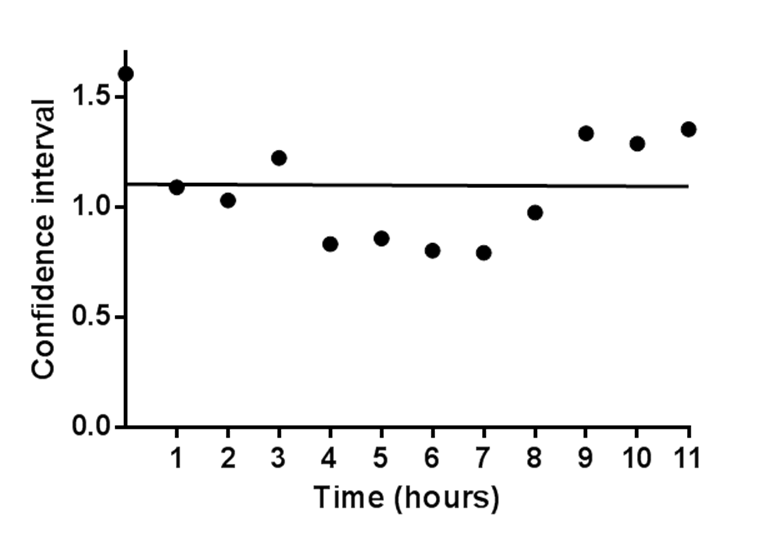

Supplement: Figure S3 — Variation of the error value (Sx) over time. The confidence interval as determined in fig. 3A was plotted against the post mortem interval. (DOCX) [file pone.0056507.s003.docx]
