# Supplementary material for: Profiling of RNA Degradation for Estimation of Post Morterm Interval
Source: PLoS One. 2013 Feb 20;8(2):e56507. doi: 10.1371/journal.pone.0056507 (PMC3577908; doi:10.1371/journal.pone.0056507)
Supplement: Table S2 — Repeatability (intra-assay variation) of qPCR measurements. (DOC) [file pone.0056507.s005.doc]

**Supplemetal Data Table S2.** Repeatability (intra-assay variation) of qPCR measurements

**Heart**

| **Gene** | **Cq** | | | **n** |
| --- | --- | --- | --- | --- |
|  | **Mean** | **SD** | **S (%)** |  |
| ***Tpm1*** | 23.9 | 0.084 | 0.355 | 6 |
| ***Alb*** | 27.21 | 0.071 | 0.259 | 6 |
| ***Actb*** | 18.64 | 0.055 | 0.298 | 6 |
| ***Gapdh*** | 14.96 | 0.035 | 0.236 | 6 |
| ***Hprt*** | 23.34 | 0.862 | 3.696 | 6 |
| ***Ppia*** | 20.83 | 0.219 | 1.052 | 6 |
| ***Srp72*** | 23.92 | 0.240 | 1.005 | 6 |
| ***Rps29*** | 26.40 | 0.035 | 0.133 | 6 |
| ***Cyp2E1*** | 24.4 | 0.353 | 1.448 | 6 |
| ***Mylk*** | 29.70 | 0.261 | 0.881 | 6 |

**Femoral quadriceps**

| **Gene** | **Cq** | | | **n** |
| --- | --- | --- | --- | --- |
|  | **Mean** | **SD** | **S (%)** |  |
| ***Tpm1*** | 25.36 | 0.502 | 1.979 | 6 |
| ***Alb*** | 20.56 | 0.473 | 2.303 | 6 |
| ***Actb*** | 21.08 | 0.077 | 0.368 | 6 |
| ***Gapdh*** | 13.49 | 0.077 | 0.576 | 6 |
| ***Hprt*** | 22.39 | 0.021 | 0.094 | 6 |
| ***Ppia*** | 21.33 | 0.014 | 0.066 | 6 |
| ***Srp72*** | 23.67 | 0.388 | 1.642 | 6 |
| ***Rps29*** | 26.49 | 0.035 | 0.133 | 6 |
| ***Cyp2E1*** | 24.26 | 0.083 | 0.343 | 6 |
| ***Mylk*** | 29.32 | 0.254 | 0.868 | 6 |

**Liver**

| **Gene** | **Cq** | | | **n** |
| --- | --- | --- | --- | --- |
|  | **Mean** | **SD** | **S (%)** |  |
| ***Tpm1*** | 22.73 | 0.070 | 0.311 | 6 |
| ***Alb*** | 13.57 | 0.289 | 2.135 | 6 |
| ***Actb*** | 17.81 | 0.056 | 0.317 | 6 |
| ***Gapdh*** | 17.22 | 0.091 | 0.532 | 6 |
| ***Hprt*** | 20.58 | 0.180 | 0.875 | 6 |
| ***Ppia*** | 19.8 | 0.028 | 0.142 | 6 |
| ***Srp72*** | 21.73 | 0.289 | 1.333 | 6 |
| ***Rps29*** | 26.58 | 0.140 | 0.527 | 6 |
| ***Cyp2E1*** | 15.34 | 0.021 | 0.138 | 6 |
| ***Mylk*** | 24.21 | 0.071 | 0.292 | 6 |
| ***Bhmt*** | 27.77 | 0.289 | 1.044 | 6 |

SD=standard deviation; S%=SD x 100/mean
