# Supplementary material for: Profiling of RNA Degradation for Estimation of Post Morterm Interval
Source: PLoS One. 2013 Feb 20;8(2):e56507. doi: 10.1371/journal.pone.0056507 (PMC3577908; doi:10.1371/journal.pone.0056507)
Supplement: Table S3 — ΔCq values of each gene for heart samples of 11 h kinetic normalized against reference gene RPS29. (DOCX) [file pone.0056507.s006.docx]

Supplemental Data Table S3. ΔCq values of each gene for heart samples of 11h kinetic normalized against reference gene RPS29.

|  | *Tpm1* | *Alb* | *Actb* | *Gapdh* | *Hprt* | *Ppia* | *Srp72* | *Cyp2E1* | *Mylk* |
| --- | --- | --- | --- | --- | --- | --- | --- | --- | --- |
| 0h | -0.775 | -0.071 | -5.021 | -6.2 | -2.381 | 0.251 | -0.433 | 0.685 | 5.523 |
| 1h | -1.375 | -0.448 | -5.91 | -8.676 | -4.028 | -4.328 | -2.84 | 0.375 | 3.491 |
| 2h | -1.03 | -0.695 | -6.348 | -8.836 | -4.283 | -4.073 | -2.971 | -0.366 | 2.843 |
| 3h | -1.913 | 1.308 | -5.356 | -7.94 | -3.861 | -4.066 | -2.356 | 1.353 | 4.081 |
| 4h | -1.848 | 0.898 | -5.19 | -8.276 | -4.25 | -5.146 | -2.445 | 1.415 | 3.701 |
| 5h | -1.93 | 1.141 | -5.666 | -7.738 | -3.86 | -5.081 | -2.16 | -0.181 | 4.266 |
| 6h | -1.303 | 1.27 | -4.908 | -7.25 | -3.191 | -3.95 | -1.111 | 3.468 | 5.09 |
| 7h | -1.641 | 2.071 | -5.691 | -8.046 | -3.85 | -4.978 | -2.57 | 1.97 | 3.88 |
| 8h | -1.043 | 1.76 | -4.426 | -6.69 | -3.246 | -4.156 | -1.47 | 1.65 | 5.19 |
| 9h | -2.143 | 2.618 | -5.305 | -7.236 | -3.713 | -4.56 | -2.103 | 1.148 | 4.83 |
| 10h | -1.645 | 0.735 | -5.415 | -7.508 | -3.733 | -4.265 | -2.011 | 0.488 | 5.035 |
| 11h | -2.59 | 0.981 | -4.78 | -6.741 | -2.431 | -1.831 | -1.056 | 1.575 | 5.47 |
