# Supplementary material for: Profiling of RNA Degradation for Estimation of Post Morterm Interval
Source: PLoS One. 2013 Feb 20;8(2):e56507. doi: 10.1371/journal.pone.0056507 (PMC3577908; doi:10.1371/journal.pone.0056507)
Supplement: Table S4 — ΔCq values of each gene for femoral quadriceps samples of 11 h kinetic normalized against reference gene RPS29. (DOCX) [file pone.0056507.s007.docx]

Supplemental Data Table S4. ΔCq values of each gene for femoral quadriceps samples of 11h kinetic normalized against reference gene RPS29.

|  | *Tpm1* | *Alb* | *Actb* | *Gapdh* | *Hprt* | *Ppia* | *Srp72* | *Cyp2E1* | *Mylk* |
| --- | --- | --- | --- | --- | --- | --- | --- | --- | --- |
| 0h | -3.013 | -2.828 | -4.225 | -12.726 | -4.443 | -5.6 | -3.291 | -3.341 | 1.297 |
| 1h | -1.77 | -1.293 | -3.78 | -11.991 | -3.018 | -4.52 | -2.745 | -2.718 | 2.166 |
| 2h | -0.943 | -0.8 | -3.563 | -11.676 | -3.223 | -4.688 | -2.888 | -0.91 | 2.271 |
| 3h | -2.508 | 0.003 | -4.165 | -11.698 | -3.368 | -5.195 | -2.786 | -4.936 | 1.927 |
| 4h | -2.488 | 1.105 | -3.608 | -11.073 | -2.225 | -4.245 | -1.94 | -2.736 | 2.406 |
| 5h | -0.653 | 0.933 | -2.958 | -10.753 | -1.325 | -3.603 | -1.301 | -1.078 | 3.48 |
| 6h | -1.463 | 1.44 | -3.246 | -11.131 | -2.385 | -4.436 | -1.435 | -1.28 | 3.433 |
| 7h | -1.943 | 1.035 | -3.233 | -11.09 | -2.178 | -3.846 | -1.823 | 0.741 | 2.448 |
| 8h | -0.426 | 1.325 | -2.98 | -10.223 | -1.236 | -3.391 | -1.086 | -1.265 | 3.087 |
| 9h | -1.283 | 2.631 | -2.775 | -9.821 | -0.866 | -2.723 | -0.291 | -0.385 | 4.091 |
| 10h | -2.808 | 1.29 | -2.828 | -9.631 | -1.451 | -3.113 | -0.053 | -1.235 | 3.821 |
| 11h | -1.788 | 1.635 | -2.333 | -9.74 | -1.105 | -2.788 | -0.503 | -0.83 | 3.237 |
