# Supplementary material for: Profiling of RNA Degradation for Estimation of Post Morterm Interval
Source: PLoS One. 2013 Feb 20;8(2):e56507. doi: 10.1371/journal.pone.0056507 (PMC3577908; doi:10.1371/journal.pone.0056507)
Supplement: Table S5 — ΔCq values of each gene for liver samples of 11 h kinetic normalized against reference gene RPS29. (DOCX) [file pone.0056507.s008.docx]

Supplemental Data Table S5. ΔCq values of each gene for liver samples of 11h kinetic normalized against reference gene RPS29.

|  | *Tpm1* | *Alb* | *Actb* | *Gapdh* | *Hprt* | *Ppia* | *Srp72* | *Cyp2E1* | *Mylk* | *Tpm1* |
| --- | --- | --- | --- | --- | --- | --- | --- | --- | --- | --- |
| 0h | -3.568 | -13.585 | -7.366 | -9.216 | -2.875 | -7.023 | 0.993 | -3.905 | -10.856 | -1.495 |
| 1h | -2.626 | -12.773 | -7.138 | -8.481 | -2.82 | -6.63 | 2.888 | -2.475 | -9.755 | -1.788 |
| 2h | -2.556 | -12.431 | -7.141 | -8.445 | -4.15 | -6.468 | 2.97 | -3.58 | -9.975 | -1.946 |
| 3h | -2.215 | -12.343 | -7.096 | -8.208 | -3.025 | -6.188 | 2.655 | -2.74 | -9.431 | -1.24 |
| 4h | -2.735 | -12.336 | -7.148 | -8.298 | -3.317 | -6.35 | 1.795 | -2.881 | -9.361 | -1.426 |
| 5h | -2.741 | -12.311 | -6.873 | -8.358 | -2.825 | -6.648 | 2.42 | -2.966 | -9.475 | -1.315 |
| 6h | -2.255 | -11.041 | -6.59 | -7.783 | -3.692 | -5.696 | 2.893 | -2.233 | -8.65 | -0.405 |
| 7h | -2.893 | -11.96 | -6.843 | -8.24 | -3.22 | -6.113 | 3.976 | -2.498 | -9.271 | -1.153 |
| 8h | -2.366 | -11.413 | -6.2 | -7.35 | -1.647 | -5.021 | 5.613 | -1.873 | -8.663 | -0.675 |
| 9h | -2.445 | -11.42 | -6.295 | -7.725 | -4.155 | -6.015 | 3.656 | -2.583 | -8.67 | -0.411 |
| 10h | -1.645 | -9.8 | -4.855 | -6.466 | -1.425 | -4.206 | 5.166 | -0.308 | -6.828 | 0.82 |
| 11h | -1.75 | -9.778 | -4.463 | -6.256 | -3.04 | -3.89 | 6.113 | -0.301 | -7.055 | 1.015 |
