# Supplementary material for: Profiling of RNA Degradation for Estimation of Post Morterm Interval
Source: PLoS One. 2013 Feb 20;8(2):e56507. doi: 10.1371/journal.pone.0056507 (PMC3577908; doi:10.1371/journal.pone.0056507)
Supplement: Table S6 — qPCR target information: Description of the genes, primer and amplicons. (DOC) [file pone.0056507.s009.doc]

**Suplemental Data Table S6.**

**qPCR target information:** Description of the genes, primer and amplicons.

| **Gene**  **symbol** | **Primer/probe sequences**  **(5’---3’)** | **Amplicon**  **length (bp)** |
| --- | --- | --- |
| ***Tpm1*** | **F:** GGTGATTGAAAATCGGGCTCT  R: GCAATGTGCTTTGCTTCCTTTAG | **82** |
| ***Alb*** | **F:** CAAGAGTGAGATCGCCCATCG  R: TTACTTCCTGCACTAATTTGGCA | **186** |
| ***Actb*** | **F:** GTGACGTTGACATCCGTAAAGA  R: GCCGGACTCATCGTACTCC | **274** |
| ***Gapdh*** | F: CTGGTGCTGCCAAGGCTGTG  R: TTCTCCAGGCGGCACGTCAG | **121** |
| ***Hprt*** | **F: CCTAAGATGAGCGCAAGTTGAA**  **R: CCACAGGACTAGAACACCTGCTAA** | **86** |
| ***Ppia*** | **F: CATCCTAAAGCATACAGGTCCTG**  **R: TTCCATGGCTTCCACAATGT** | **212** |
| ***Bhmt*** | **F: GCGTGAGCCAGACGCCTTCATA**  **R: CCTTTCTGGGGCCAACTCCTCT** | **128** |
| ***Srp72*** | **F: CACCCAGCAGACAGACAAACTG**  **R: GCACTCATCGTAGCGTTCCA** | **81** |
| ***Rps29*** | **F: GCACTGCTGAGAGCAAGATG**  **R: ACCCATCTTGCCTTCAGACG** | **78** |
| ***Cyp2E1*** | **F: CCACGATGCGCCTCTGA**  **R: GATATTCCCAAGTCTTTAACCA** | **165** |
| ***Mylk*** | **F: AATCTCATTCCCGTCGTGAAGCCA**  **R: AAGTCATGGATGGAAGCCAGGTCA** | **181** |
