# Supplementary material for: Profiling of RNA Degradation for Estimation of Post Morterm Interval
Source: PLoS One. 2013 Feb 20;8(2):e56507. doi: 10.1371/journal.pone.0056507 (PMC3577908; doi:10.1371/journal.pone.0056507)
Supplement: Table S7 — qPCR Validation. Standard curves were generated either from cDNAs (for all RNAs) setting the undiluted sample as 1 arbitrary unit. The Cq values were calculated automatically by the iQ5 Optical system software, version 2 using the “second derivative maximum” method. Standard curves: the PCR-efficiency (E = 10−1/slope) and the slope, intercept, and error of the regression line as well as the so-called dynamic range and the Cq variation at the lower limit (the endpoint of the dynamic range) were calculated by the iQ5 Optical system software. The presented data was retrieved from heart samples except for BHMT, which was from liver tissue sample. (DOC) [file pone.0056507.s010.doc]

**Supplemental Data Table S7. qPCR Validation**

Standard curves were generated either from cDNAs (for all RNAs) setting the undiluted sample as 1 arbitrary unit. The Cq values were calculated automatically by the iQ5 Optical system software, version 2 using the “second derivative maximum” method.

Standard curves: the PCR-efficiency (E=10^-1/slope^) and the slope, intercept, and error of the regression line as well as the so-called dynamic range and the Cq variation at the lower limit (the endpoint of the dynamic range) were calculated by the iQ5 Optical system software.

The presented data was retrieved from heart samples except for BHMT, which was from liver tissue sample.

| **Gene** | **PCR-Efficiency** | **Slope** | **y-Intercept** | **Error** | **Dynamic range** | **Ct variationat lower limit (SD)** |
| --- | --- | --- | --- | --- | --- | --- |
| ***Tpm1*** | 1.898 | -3.593 | 23.59 | 0.0135 | 21.6-33.3 | 0.98 |
| ***Alb*** | 1.921 | -3.527 | 27.12 | 0.0311 | 22.4-34.1 | 0.76 |
| ***Actb*** | 1.920 | -3.530 | 21.04 | 0.0343 | 20.0-34.5 | 0.66 |
| ***Gapdh*** | 2.012 | -3.293 | 15.57 | 0.0123 | 13.4-29.6 | 0.85 |
| ***Hprt*** | 1.966 | -3.406 | 24.41 | 0.0213 | 22.7-35.1 | 0.14 |
| ***Ppia*** | 1.985 | -3.358 | 20.47 | 0.0298 | 19.9-35.5 | 0.74 |
| ***Bhmt*** | 1.931 | -3.499 | 28.33 | 0.0091 | 23.4-35.7 | 0.52 |
| ***Srp72*** | 1.982 | -3.366 | 23.84 | 0.0043 | 22.7-38.4 | 0.12 |
| ***Rps29*** | 1.991 | -3.344 | 27.72 | 0.0412 | 22.7-33.1 | 0.45 |
| ***Cyp2E1*** | 1.875 | -3.663 | 25.12 | 0.0258 | 16.5-33.7 | 0.35 |
| ***Mylk*** | 1.963 | -3.414 | 30.29 | 0.0089 | 27.8-38.9 | 0.45 |
