# Supplementary material for: Profiling of RNA Degradation for Estimation of Post Morterm Interval
Source: PLoS One. 2013 Feb 20;8(2):e56507. doi: 10.1371/journal.pone.0056507 (PMC3577908; doi:10.1371/journal.pone.0056507)
Supplement: Supplementary file 10 [file pone.0056507.s002.docx]

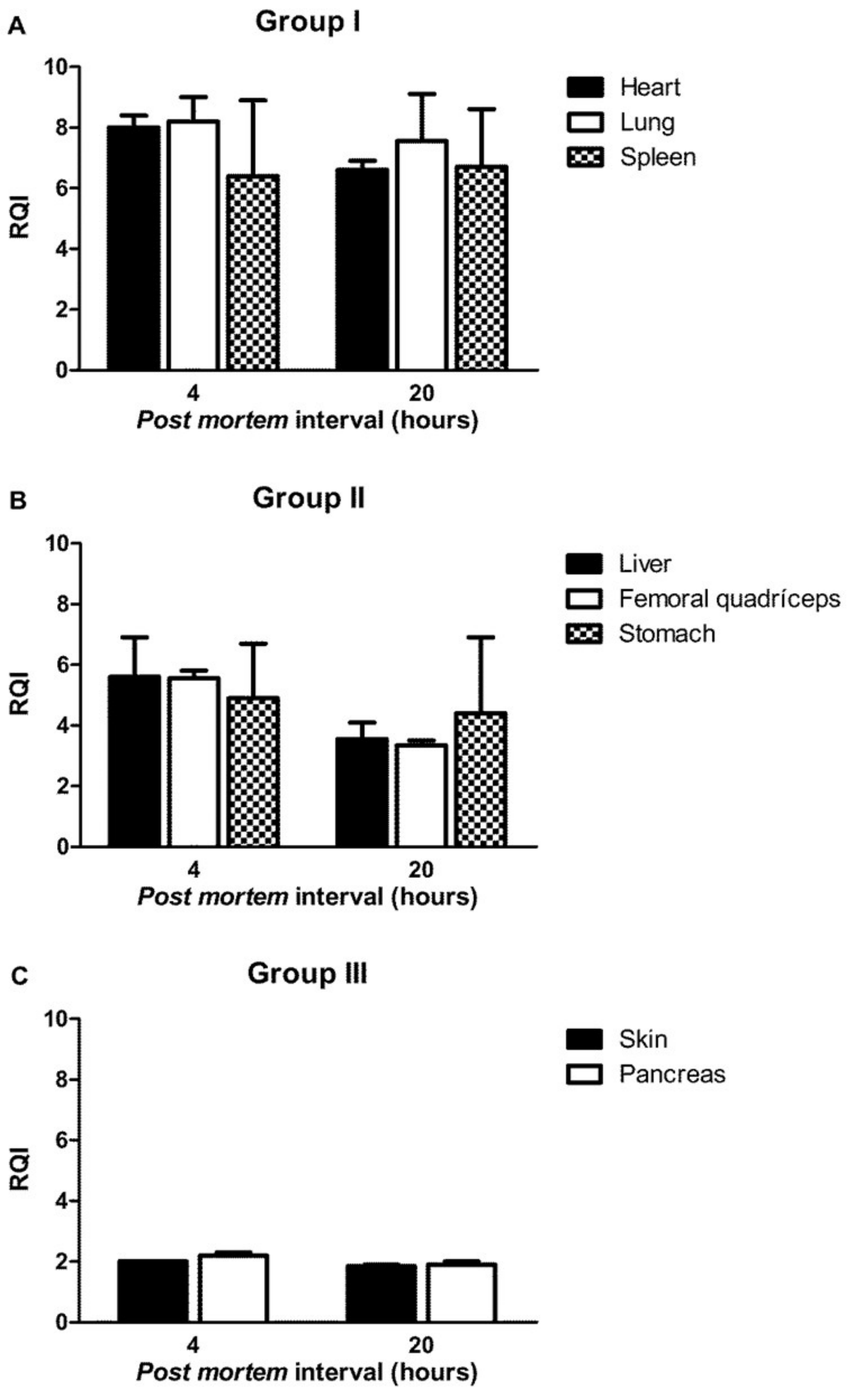
A
Group I
RQI
10
8
6
4
2
0
Heart
Lung
Spleen
4
20
Post mortem interval (hours)
B
Group II
RQI
10
8
6
4
2
0
Liver
Femoral quadríceps
Stomach
4
20
Post mortem interval (hours)
C
Group III
RQI
10
8
6
4
2
0
Skin
Pancreas
4
20
Post mortem interval (hours)
